# Integrated analysis of single-cell and bulk RNA-seq reveals MAGEA3/6-associated immune subtypes and key immune genes in gastric cancer

**DOI:** 10.1371/journal.pone.0338705

**Published:** 2025-12-26

**Authors:** Linen Li, Hao Chen, Feng Zhou, Yanqing Shi

**Affiliations:** 1 Department of Gastroenterology, The First Affiliated Hospital of Nanchang University, Nanchang, Jiangxi, China; 2 Department of Gastroenterology, The Central Hospital of Jingmen, Jingmen, Hubei, China; 3 Department of Gastroenterology, Affiliated Hospital of Jiujiang University, Jiujiang, Jiangxi, China; Korea Institute of Radiological and Medical Sciences, KOREA, REPUBLIC OF

## Abstract

The immune microenvironment is critical in gastric cancer (GC), yet epithelial-specific genes linked to immune infiltration remain poorly defined. We integrated single-cell RNA-seq (GSE112302) and TCGA-STAD data to identify epithelial-related differentially expressed genes (DEGs). MAGEA3 and MAGEA6 were selected to classify immune subtypes. Immune infiltration was analyzed using ESTIMATE, CIBERSORT, ssGSEA, and Xcell. WGCNA and survival analysis identified prognostic immune-related genes. MAGEA3/6-high tumors showed low immune infiltration, defining an immune-cold subtype, while MAGEA3/6-low tumors were immune-hot. Further analysis confirmed that BCL11B and PAEP expression levels were significantly associated with immune cell infiltration and immune regulatory activity. MAGEA3/6 expression defines immune subtypes in GC and highlights potential targets for immunotherapy.

## 1. Introduction

Gastric cancer (GC) remains one of the leading causes of cancer-related death worldwide, characterized by high heterogeneity and poor prognosis [1]. Despite recent advances in immunotherapy, only a subset of GC patients responds favorably to such treatments [2], underscoring the need for precise molecular stratification and identification of novel immune-related biomarkers.

The tumor immune microenvironment (TIME) plays a pivotal role in tumor progression, immune evasion, and therapeutic resistance [3,4]. Increasing evidence indicates that tumor epithelial cells not only serve as the origin of malignancy but also actively shape the local immune landscape through cytokine secretion, antigen presentation, and immune checkpoint expression [5,6]. The MAGEA family (Melanoma Antigen Gene A family) is an important subgroup of cancer-testis antigens (CTAs), which are widely expressed in various tumor tissues but are normally restricted to immune-privileged sites such as the testis in healthy tissues [7]. In recent years, an increasing number of studies have revealed that MAGEA family genes are closely associated with tumor immune infiltration and may play critical roles in immune evasion and the remodeling of the tumor immune microenvironment [8,9].

Single-cell RNA sequencing (scRNA-seq) technology enables the high-resolution dissection of cell-type–specific gene expression in complex tissues [10,11]. When integrated with bulk transcriptomic data from large-scale cancer cohorts such as The Cancer Genome Atlas (TCGA), scRNA-seq provides powerful insights into cellular heterogeneity and potential prognostic markers.

In this study, we integrated scRNA-seq data from GSE112302 and bulk RNA-seq data from TCGA-STAD to identify epithelial-specific DEGs associated with immune infiltration in GC. We focused on the MAGEA family genes, particularly MAGEA3 and MAGEA6, which were found to stratify GC into immune-inflamed and immune-excluded subtypes. Through comprehensive immune infiltration analysis and co-expression network modeling, we further identified key immune regulatory genes with prognostic significance. Our findings provide new perspectives on tumor-epithelial–immune crosstalk and offer potential biomarkers for immunotherapy stratification in gastric cancer.

## 2. Methods

### 2.1 Data sources

We analyzed scRNA-seq data from GEO (GSE112302), comprising single cells from 6 GC tumors and 4 adjacent normals (707 cells total), and TCGA-STAD dataset: This dataset includes 410 primary gastric adenocarcinoma tissues and 36 adjacent normal tissues obtained from The Cancer Genome Atlas (TCGA) database. Available clinical variables included survival time, status, gender, age and tumor stage. GSE84437 dataset: The GSE84437 cohort comprises 483 gastric cancer samples profiled on the GPL6947 platform (Illumina HumanHT-12 V3.0 expression beadchip). Detailed clinical annotations include survival time, status, gender, age and tumor stage. GSE29272 dataset: The GSE29272 dataset contains 134 gastric cancer tissues and 134 matched non-tumor mucosae, measured on the GPL96 platform ([HG-U133A] Affymetrix Human Genome U133A Array). The overall tissue categories consist of gastric tumors from both cardia and non-cardia regions, along with normal gastric glands. A waterfall plot was generated using the R package ComplexHeatmap [12] to illustrate the gene mutation profiles of STAD patients. The raw data from GSE112302, GSE84437,GSE29272 and TCGA gastric cancer datasets were normalized using the “limma” package [13].

### 2.2 scRNA-seq processing

After obtaining transcripts-per-million (TPM) matrices, we performed quality control by filtering cells with low gene counts or high mitochondrial gene content (following established protocols). Data were normalized and log-transformed using the Seurat package [14,15]. We performed principal component analysis (PCA) [16] on the top variable genes, followed by K-nearest-neighbor graph construction and clustering (FindNeighbors, FindClusters) to identify cell clusters. Clusters were annotated based on SingleR, isolating epithelial cell clusters for downstream analysis. Within the epithelial clusters, we compared gene expression between tumor-derived and normal-derived epithelial cells. DEGs were identified using the Wilcoxon rank-sum test, with thresholds |log2FC| > 2 and adjusted p < 0.05.

### 2.3 Bulk RNA-seq DE and intersection

In parallel, TCGA-STAD tumor and normal samples were compared using the DEseq2 [17] package to identify DEGs (adjusted p < 0.05, |log2FC| > 2). We intersected the scRNA-derived epithelial DEGs with bulk DEGs, yielding 23 overlapping epithelial-related DEGs for further analysis.

### 2.4 Subtype clustering by MAGEA3/6

We calculated an immune score for each TCGA sample using the ESTIMATE algorithm. Spearman correlation between each of the 23 DEGs and immune score was assessed; MAGEA3 and MAGEA6 showed the strongest negative correlations. We used consensus clustering on TCGA-STAD expression data [18], using MAGEA3 and MAGEA6 as features, to partition samples into two groups (group1: high MAGEA3/6; group2: low MAGEA3/6). The optimal cluster number was chosen via silhouette analysis.

### 2.5 Functional enrichment analysis

Gene Ontology (GO), Kyoto Encyclopedia of Genes and Genomes (KEGG) pathway, and Gene set enrichment analysis (GSEA) was performed using the R package clusterProfiler [18] to identify significant functional differences between the two groups. Pathways were considered significantly enriched based on the following criteria: normalized enrichment score (|NES| > 1), P value < 0.05, and FDR-adjusted q value < 0.05.

### 2.6 Immune infiltration analysis

ESTIMATE is a method based on gene expression profiles of tumor samples to estimate the proportions of stromal and immune cells. This method was used to assess the tumor microenvironment (TME) of each STAD patient. Using the R package estimate [19], stromal score (stromal content), immune score (extent of immune cell infiltration), ESTIMATE score (a composite index of stromal and immune components), and tumor purity were calculated.

CIBERSORT is a method that infers cell composition from gene expression profiles. This deconvolution algorithm was used to estimate the proportions of 22 immune cell types in each STAD patient [20]. The total proportion of the 22 immune cell types in each sample sums to 1.

Single-sample gene set enrichment analysis (ssGSEA) method from the R package GSVA [21] was used to calculate the infiltration levels of 28 immune cell types based on the expression profiles of genes from 28 published immune cell gene sets [22].

Using the single-sample gene set enrichment analysis method from the R package Xcell, the infiltration levels of 64 immune cell types were calculated based on the expression levels of gene sets for 64 published immune cell types [23].

### 2.7 Weighted gene co-expression network analysis

Using TCGA-STAD expression data, we constructed a signed co-expression network with the WGCNA package [24]. Modules of co-expressed genes were identified, and module eigengenes were correlated with immune scores and subtype membership. The yellow module, containing 506 genes, showed highest association with immune score.

### 2.8 Survival analysis of module genes

Within the yellow module, we performed univariate Cox proportional hazards regression (and log-rank test) for each gene against overall survival in TCGA-STAD and GSE84437. Genes with hazard ratio (HR) < 1 (protective) or > 1 (risk) and p < 0.05 were noted. We focused on CTLA4, TAP1, BCL11B (protective) and PAEP (risk) for further analysis. Their expression correlations with MAGEA3/6 and immune scores were evaluated.

### 2.9 Statistical analysis

All statistical analyses were carried out using R and SPSS software. Figures were compiled using Adobe Illustrator. The Wilcoxon rank-sum test was applied for box plot comparisons. Spearman’s correlation coefficient was used to assess correlations between variables. Univariate and multivariate Cox proportional hazards regression models were employed to determine the significance of prognostic factors. Survival analysis was conducted using the Kaplan–Meier method, with differences between groups assessed via the log-rank test. All tests were two-sided, and a p-value less than 0.05 was considered statistically significant.

## 3. Results

### 3.1 Identification of epithelial differentially expressed genes by single-cell RNA sequencing

**Fig 1:**
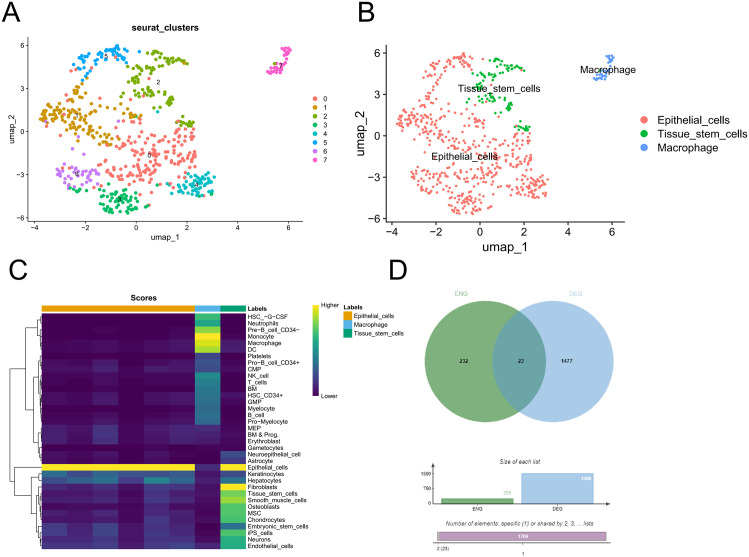
Identification of epithelial cells and epithelial differentially expressed genes (DEGs) in gastric tissues. **(A)** Principal component analysis (PCA) and graph-based clustering of single-cell RNA sequencing data identified eight distinct cell clusters. **(B)** Uniform Manifold Approximation and Projection (UMAP) plot showing annotated cell types based on canonical marker genes. **(C)** Expression of canonical marker genes used for cell type identification. **(D)** Venn diagram showing the intersection of DEGs between tumor vs. normal epithelial cells and TCGA-STAD dataset, resulting in 23 epithelial DEGs.

We first performed quality control on the single-cell RNA sequencing dataset GSE112302 (S1 Fig). A total of 401 tumor tissue cells and 306 normal tissue cells were subjected to principal component analysis (PCA) and subsequently clustered into eight distinct groups using a graph-based clustering approach (Fig 1A). Next, we annotated the cell types and identified clusters corresponding to epithelial cells, tissue stem cells, and macrophages, based on the expression of canonical marker genes (Fig 1B and 1C). We then performed differential expression analysis between tumor epithelial cells and normal epithelial cells, and intersected the resulting genes with those differentially expressed in TCGA. A total of 23 genes were identified as epithelial differentially expressed genes (Fig 1D).

### 3.2 Identification of epithelial-related DEGs with immune infiltration

**Fig 2:**
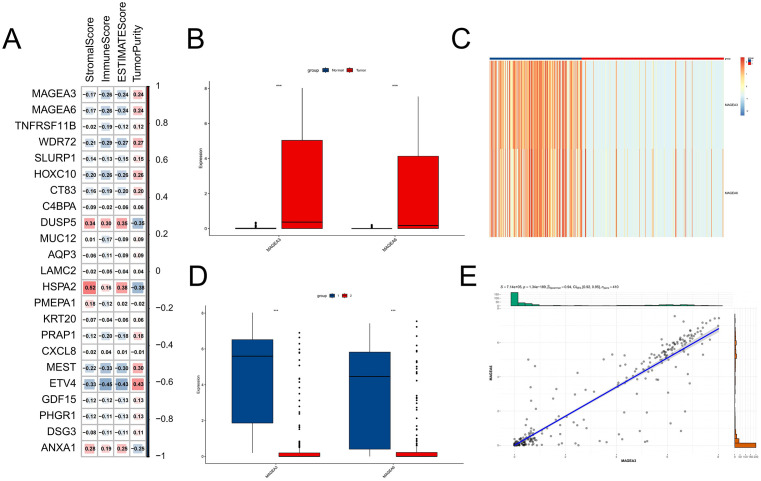
Identification of epithelial-related DEGs associated with immune infiltration. **(A)** Correlation analysis between 23 epithelial-related DEGs and immune scores calculated using the ESTIMATE algorithm; MAGEA3 and MAGEA6 show strongest correlations. **(B)** Boxplots comparing MAGEA3 and MAGEA6 expression between tumor and adjacent normal tissues in TCGA-STAD. **(C)** Consensus clustering of 410 TCGA-STAD samples based on MAGEA3 and MAGEA6 expression, revealing two molecular subtypes. **(D)** Expression levels of MAGEA3 and MAGEA6 in Group 1 (high expression) and Group 2 (low expression). **(E)** Spearman correlation analysis between MAGEA3 and MAGEA6 expression levels.

To investigate the involvement of Epithelial-related DEGs in tumor immunity of stomach adenocarcinoma (STAD) patients, we first used the ESTIMATE algorithm to calculate four immune-related scores for each sample, reflecting the relative abundance of stromal and immune components in the tumor microenvironment. Among 23 Epithelial-related DEGs, MAGEA3 and MAGEA6 demonstrated the strong absolute correlations with immune scores and were thus selected for downstream analyses (Fig 2A). Based on RNA-seq data from TCGA-STAD, tumor tissues exhibited significantly elevated MAGEA3 and MAGEA6 expression compared to normal tissues (Fig 2B). Using the expression profiles of MAGEA3 and MAGEA6, we carried out consensus clustering on 410 STAD samples from TCGA, resulting in two molecular subgroups (Fig 2C and S2 Fig). group 1 (n = 161) was characterized by high MAGEA3 and MAGEA6 expression, whereas group 2 (n = 249) exhibited the inverse pattern—lower MAGEA3 and MAGEA6 expression (Fig 2D). Since the expression patterns of these two genes were consistent, we further investigated their correlation and found a significant positive Spearman correlation between them (R = 0.94, P = 1.34e-189; Fig 2E).

### 3.3 Functional enrichment analysis of DEGs between group1 and group2

**Fig 3:**
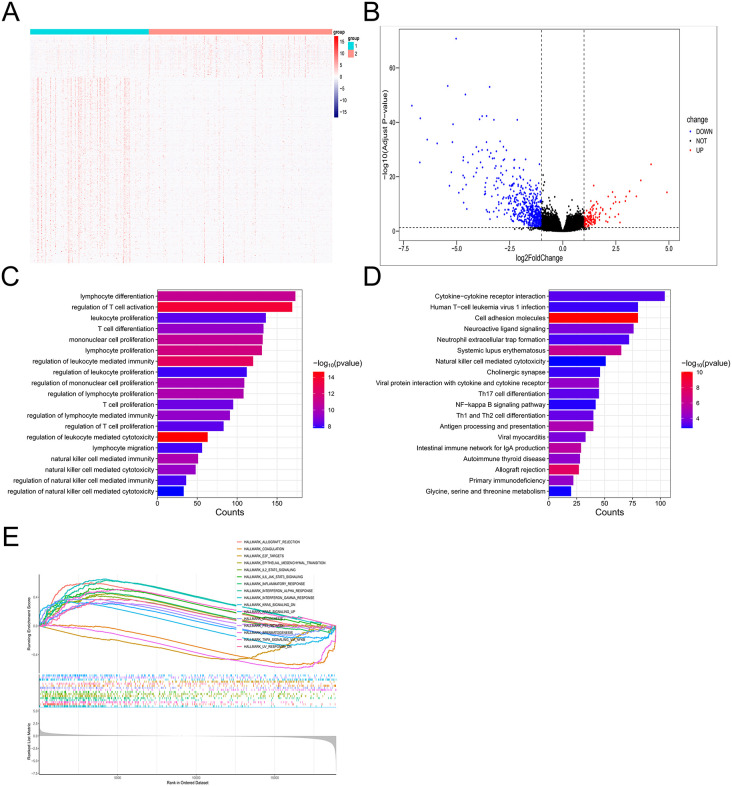
Functional enrichment analysis of DEGs between Group 1 and Group 2. **(A)** Heatmap of DEGs shows clear separation between Group 1 and Group 2. **(B)** Volcano plot of DEGs between Group 1 and Group 2. **(C)** GO enrichment analysis of DEGs. **(D)** KEGG pathway analysis of DEGs. **(E)** Gene Set Enrichment Analysis (GSEA) of Hallmark gene sets showing enrichment of immune/inflammatory pathways.

The heatmap illustrates the global gene expression patterns, clearly separating the samples into two groups (group1 and group2), indicating substantial transcriptional divergence (Fig 3A). The volcano plot shows the distribution of differentially expressed genes, highlighting a large number of significantly differentially expressed genes (Fig 3B). The GO enrichment bar plot reveals that many of these differentially expressed genes are significantly associated with immune-related biological processes, including lymphocyte differentiation, regulation of T cell activation, leukocyte proliferation, and natural killer cell mediated immunity (Fig 3C). The pathway enrichment bar plot shows KEGG pathways and disease associations enriched among the differentially expressed genes. These include Cytokine−cytokine receptor interaction, Th17 cell differentiation, NF−kappa B signaling pathway and Th1 and Th2 cell differentiation (Fig 3D). The GSEA enrichment presents Hallmark gene set enrichment analysis results. Immune and inflammation-related gene sets such as allograft rejection, interferon alpha and gamma response, IL2/STAT5/signaling, IL6/JAK-STAT3 signaling, and TNF-α/NF-κB signaling are enriched (Fig 3E). In summary, these results indicate that the differentially expressed genes between group 1 and group 2 are significantly associated with immune infiltration.

### 3.4 Comparison of immune infiltration

**Fig 4:**
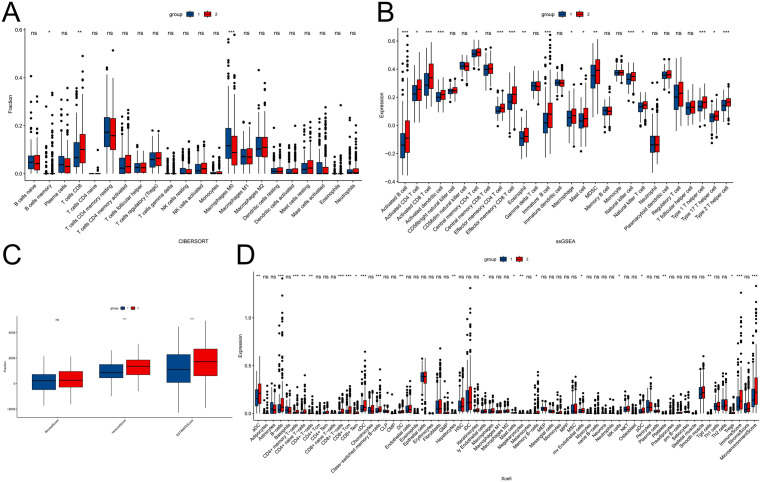
Comparison of immune infiltration between the two molecular subtypes. **(A)** CIBERSORT analysis showing relative immune cell proportions. **(B)** ssGSEA scores of 28 immune cell types. **(C)** ESTIMATE-derived stromal, immune, and ESTIMATE scores. **(D)** Xcell analysis of 64 immune cell types.

To further explore the differences in immune function between the two subtypes, we performed ESTIMATE, CIBERSORT, Xcell, and ssGSEA analyses. The ESTIMATE results showed that group 1 had significantly lower stromal scores, immune scores, and overall ESTIMATE scores compared to group 2 (Fig 4C). In the CIBERSORT analysis, the proportion of CD8 ⁺ T cells were notably lower in group 1 (Fig 4A). Moreover, ssGSEA revealed that group 1 exhibited lower enrichment levels across multiple immune cell subtypes, including activated B cells, CD4 ⁺ T cells, CD8 ⁺ T cells, natural killer cells, and natural killer T cells (Fig 4B). Consistently, the Xcell analysis also indicated lower enrichment of B cells, CD4 ⁺ T cells, and CD8 ⁺ T cells in group 1 (Fig 4D). A heatmap was generated to display the overall distribution of 28 immune cell subtypes between the two groups (S3 Fig). In summary, these results indicate that group 1 shows significantly reduced immune infiltration compared to group 2, especially in CD8 ⁺ T cells. To evaluate the potential response of STAD patients to immunotherapy, we examined several key immune checkpoint targets (PDCD1, CD274, and PDCD1LG2) and compared their expression levels between the two subtypes. The results showed that PDCD1 and CD274 were upregulated in group 2 (S4 Fig). These findings suggest that patients in group 2 may be more sensitive to immunotherapy than those in group 1. Additionally, in the GSE29272 dataset, we divided the samples into group 1 and group 2 based on the expression levels of MAGEA3/MAGEA6 and analyzed the differences in MAGEA3 and MAGEA6 expression between the two groups, as well as the differences in immune infiltration. We also examined the differences in MAGEA3 and MAGEA6 expression between tumor tissues and adjacent normal tissues. The results showed that compared to group 1, group 2 exhibited higher expression levels of both MAGEA3 and MAGEA6 (S5A-S5B Fig). The differences in immune infiltration between the two groups were primarily enriched in CD4 + memory T cells, Th1 cells, and Th2 cells (S5C Fig), among others. Additionally, the expression of MAGEA3 and MAGEA6 was higher in tumor tissues compared to adjacent normal tissues (S5D Fig).

### 3.5 WGCNA and Identification of Hub Genes Related With Immunity

**Fig 5:**
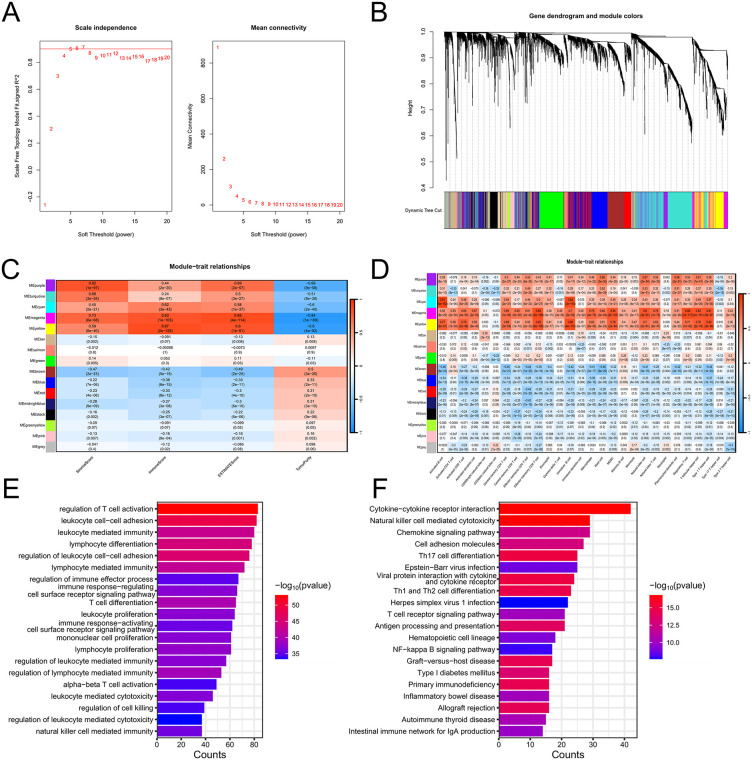
Weighted gene co-expression network analysis (WGCNA) identifies immune-related gene modules. **(A)** Analysis of network topology for soft powers. **(B)** Gene dendrogram and module colors. **(C-D)** Correlation between module eigengenes and immune traits. **(E)** GO enrichment analysis of yellow module genes. **(F)** KEGG pathway analysis of yellow module genes.

Subsequently, the differentially expressed genes between group 1 and group 2 were subjected to weighted gene co-expression network analysis (WGCNA) (Fig 5A and 5B). To identify modules associated with immune traits, we performed a correlation analysis between gene modules and phenotypic characteristics (Fig 5C and 5D). The results revealed that the yellow module was highly correlated with immunity (R = 0.87, P = 2e-128), and thus it was selected as the key module and the yellow module contained 506 genes. GO and KEGG enrichment analyses were performed on the genes in the yellow module, and the results indicated that these genes were primarily enriched in immune-related biological functions and pathways (Fig 5E and 5F).

### 3.6 Evaluation of clinical characteristics

**Fig 6:**
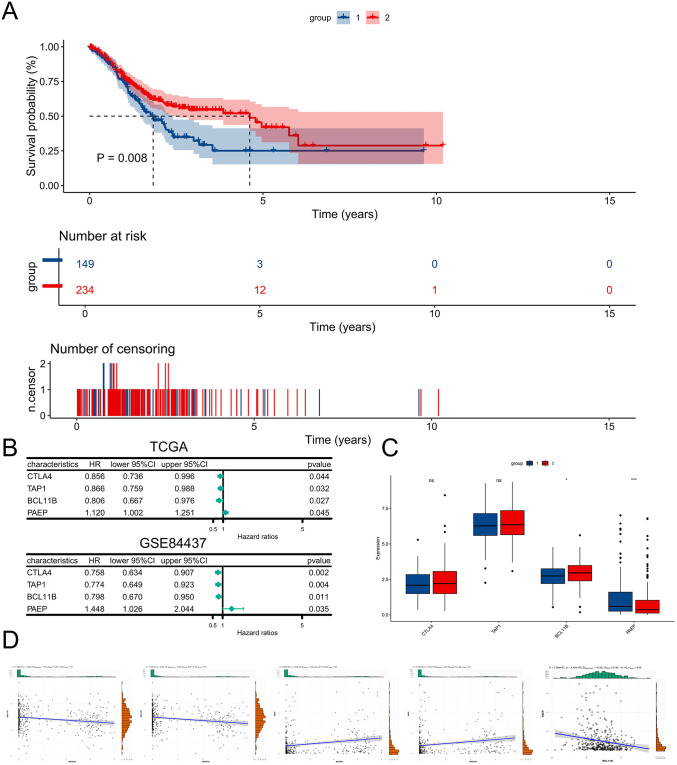
Clinical significance and mutation profiles of molecular subtypes. **(A)** Survival analysis of 2 groups. **(B)** Forest plot from Cox regression analysis identifying CTLA4, TAP1, BCL11B, and PAEP. **(C)** Boxplots comparing expression of CTLA4, TAP1, BCL11B and PAEP between Group 1 and Group 2. **(D)** Correlation analysis of MAGEA3/6 with BCL11B and PAEP expression.

To investigate the differences in clinical characteristics between the two groups, we first performed a survival analysis, which showed that group 1 had a poorer prognosis (Fig 6A). Different gene mutations can influence patient prognosis; therefore, we evaluated the mutational landscape in STAD. The results showed that group 1 exhibited a higher mutation rate of TP53 compared to group 2 (S6 Fig). Subsequently, we conducted Cox regression analysis on the genes in the yellow module using the TCGA and GSE84437 datasets. The results indicated that CTLA4, TAP1, and BCL11B were protective factors, while PAEP was identified as a risk factor (Fig 6B). Next, we analyzed the expression levels of the four genes in group 1 and group 2. The results showed that, compared with group 2, BCL11B was downregulated in group 1, while PAEP was upregulated (Fig 6C). In addition, BCL11B expression was negatively correlated with the expression of MAGEA3 and MAGEA6, whereas PAEP expression was positively correlated with both MAGEA3 and MAGEA6. In addition, BCL11B and PAEP expression were also negatively correlated (Fig 6D).

### 3.7 The correlation between BCL11B and PAEP and immune infiltration

**Fig 7:**
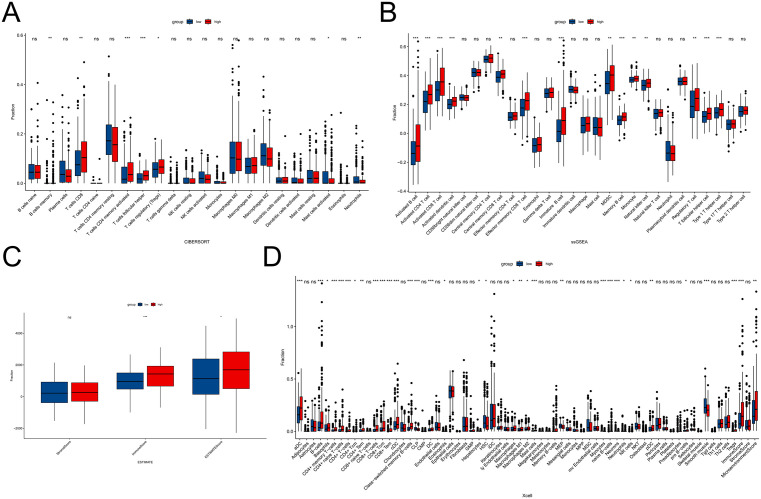
Immune infiltration analysis based on BCL11B expression levels. **(A)** CIBERSORT analysis showing relative immune cell proportions. **(B)** ssGSEA scores of 28 immune cell types. **(C)** ESTIMATE-derived stromal, immune, and ESTIMATE scores. **(D)** Xcell analysis of 64 immune cell types.

**Fig 8:**
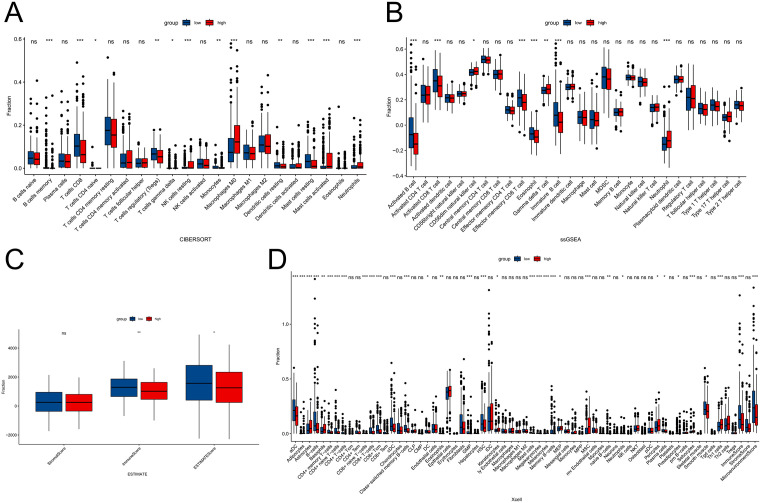
Immune infiltration analysis based on PAEP expression levels. **(A)** CIBERSORT analysis showing relative immune cell proportions. **(B)** ssGSEA scores of 28 immune cell types. **(C)** ESTIMATE-derived stromal, immune, and ESTIMATE scores. **(D)** Xcell analysis of 64 immune cell types.

Subsequently, based on the expression levels of BCL11B and PAEP, samples were divided into high-expression and low-expression groups, respectively. The expression of immunoregulatory targets and the extent of immune infiltration were evaluated using ESTIMATE, CIBERSORT, ssGSEA, and Xcell (Fig 7A–7D and Fig 8A-8D). The results showed that the low BCL11B expression group exhibited significantly lower immune activity compared to the high BCL11B group, while the high PAEP expression group showed markedly reduced immune activity compared to the low PAEP group.

## 4. Discussion

In this study, we systematically explored the role of epithelial-related differentially expressed genes (DEGs), particularly MAGEA3 and MAGEA6, in shaping the tumor immune microenvironment (TME) of stomach adenocarcinoma (STAD). Through integrative analysis of single-cell RNA sequencing and TCGA bulk RNA-seq data, we identified 23 epithelial DEGs, among which MAGEA3 and MAGEA6 exhibited the strongest correlation with immune infiltration scores, suggesting their potential as key immunomodulatory genes. MAGEA family proteins are typically silent in normal somatic cells but overexpressed in various cancers [25], and MAGEA family genes are closely associated with tumor immune infiltration [8,9], making them prime targets for immunotherapy. In our cohort, high MAGEA3/6 expression defined an “MAGE-high” subtype (group1) of GC. Consensus clustering of TCGA-STAD samples by MAGEA3/6 expression partitioned tumors into two subtypes with markedly different immune landscapes. group1 (MAGE-high) exhibited significantly lower ESTIMATE immune scores and higher tumor purity, whereas group2 (MAGE-low) showed enriched immune and stromal signatures. This suggests group1 tumors are relatively “immune cold,” potentially more adept at immune evasion, while group2 are “immune hot.” This pattern was corroborated across multiple algorithms: CIBERSORT showed group1 had reduced infiltration of T cells, whereas group2 had higher infiltration of these effector cells. Similarly, ssGSEA and Xcell indicated group1 had lower enrichment scores for T cell, NK cell, and antigen-presentation pathways. Such dichotomies echo previous classifications of GC and other cancers into immune-inflamed versus immune-excluded subtypes. Notably, the immune-cold group1 might be less responsive to immunotherapy, paralleling observations that low-immune-score tumors often have worse prognosis and therapeutic response [26,27].

Differentially expressed genes between the two clusters were significantly enriched for immune-related pathways in GO, KEGG, and Hallmark sets. Indeed, previous work has shown MAGEA3 can alter tumor immune microenvironment and associate with immune infiltrates, suggesting it may promote immune tolerance [28].

To identify key immune regulators, WGCNA on TCGA-STAD data identified a “yellow” module of 506 genes strongly associated with immune score. This module was enriched for T-cell and antigen-presentation genes. Four genes stood out by survival analysis: CTLA4, TAP1, and BCL11B (protective; HR < 1) and PAEP (risk; HR > 1). From this module, BCL11B and PAEP emerged as critical immune regulators. Notably, BCL11B, a known transcription factor involved in T cell development [29,30], was significantly downregulated in group 1 and negatively correlated with MAGEA3/6, whereas PAEP, previously implicated in immune suppression, was upregulated and positively correlated with MAGEA3/6. Functional analyses confirmed that BCL11B high expression and PAEP low expression were both associated with enhanced immune infiltration, underscoring their importance in modulating the TME. We observed PAEP expression to be higher in the MAGE-high/immune-cold group and negatively correlated with immune scores. Literature on lung cancer and reproduction indicates PAEP/glycodelin helps tumors evade immune surveillance [31–33], which agrees with our finding that PAEP portends worse survival and an immunosuppressive TME. Overall, our integrated approach highlights a GC subtype defined by high MAGEA3/6 expression with an immune-excluded phenotype, versus a subtype with low MAGEA3/6 and active immune infiltration. The identified immune-related genes (BCL11B, PAEP) may contribute to these phenotypes. For example, high MAGEA expression could coincide with epigenetic changes that suppress antigen presentation and T-cell recruitment, lowering BCL11B while raising PAEP to blunt immunity. Conversely, the MAGE-low tumors maintain T-cell transcription (BCL11B), and downregulate PAEP supporting vigorous immune activity. Clinically, the MAGE-high/PAEP-high subtype might benefit from strategies to reverse immune exclusion, whereas the MAGE-low subtype might already be primed for checkpoint blockade. These findings not only enhance our understanding of the immunosuppressive mechanisms driven by epithelial-derived cancer/testis antigens but also suggest that MAGEA3, MAGEA6, BCL11B, and PAEP may serve as potential biomarkers or therapeutic targets for improving immunotherapy responses in STAD patients. This study has limitations. The scRNA sample size (707 cells) is modest, and further validation in larger single-cell cohorts is warranted. The analysis is correlative; mechanistic studies are needed to test whether MAGEA3/6 directly modulate the immune milieu. Moreover, TCGA-STAD has relatively few normal samples, which could affect bulk DEG results. Despite these caveats, our findings are supported by multiple algorithms and literature links, providing a cohesive picture of how epithelial gene expression, especially MAGE-A antigens, associates with immune states in GC.

## 5. Conclusion

Our comprehensive analysis reveals that MAGEA3 and MAGEA6 are epithelial-derived genes that are significantly associated with immune infiltration and tumor immune microenvironment remodeling in stomach adenocarcinoma. High expression of these genes delineates a molecular subtype with reduced immune cell infiltration, particularly CD8 ⁺ T cells, and poorer prognosis. Further investigation identified BCL11B and PAEP as key modulators of immune activity that are tightly linked to MAGEA3/6 expression.

## Supporting information

S1 Fig Quality control and clustering of single-cell RNA-seq data. (PDF)

S2 Fig Determination of optimal cluster number for consensus clustering. (PDF)

S3 Fig Immune cell subtype distribution between molecular subgroups. (PDF)

S4 Fig Evaluation of sensitivity to immunotherapy. (PDF)

S5 Fig Analysis of MAGEA3/MAGEA6 expression and immune infiltration in GSE29272 dataset. (PDF)

S6 Fig Somatic mutation landscape of STAD patients in the two subgroups. (PDF)
