# Supplementary figures and images for: Integrated analysis of single-cell and bulk RNA-seq reveals MAGEA3/6-associated immune subtypes and key immune genes in gastric cancer
Source: PLoS One. 2025 Dec 26;20(12):e0338705. doi: 10.1371/journal.pone.0338705 (PMC12742767; doi:10.1371/journal.pone.0338705)

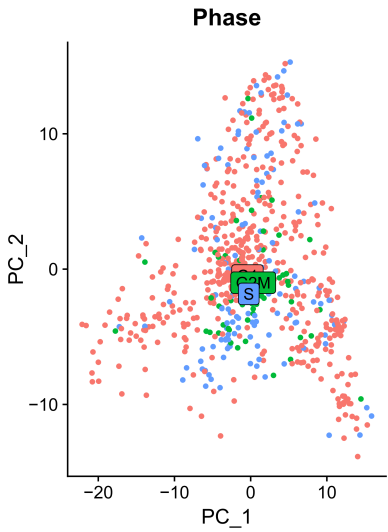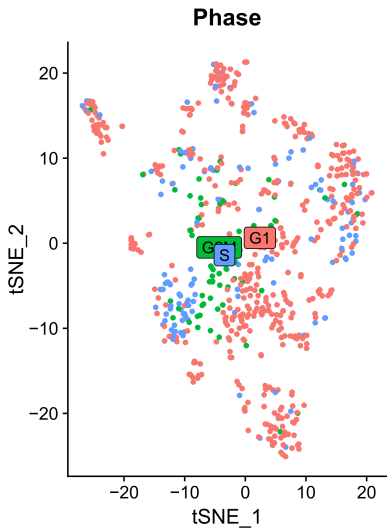

● G1 ● G2M ● S

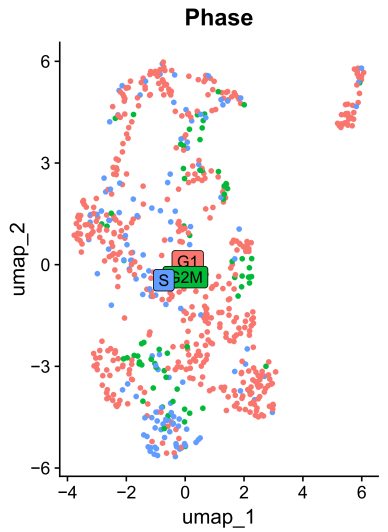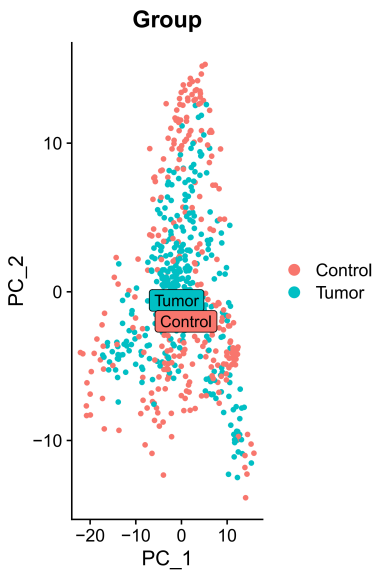

● Control  
● Tumor

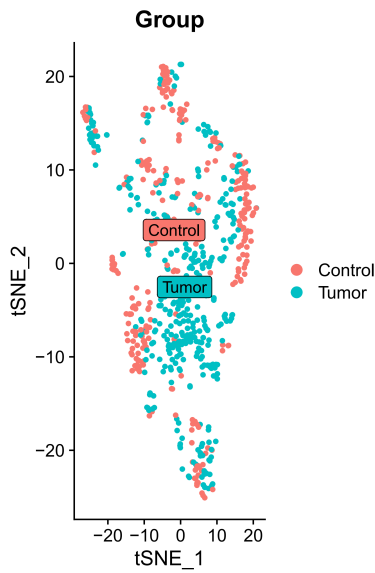

● Control  
● Tumor

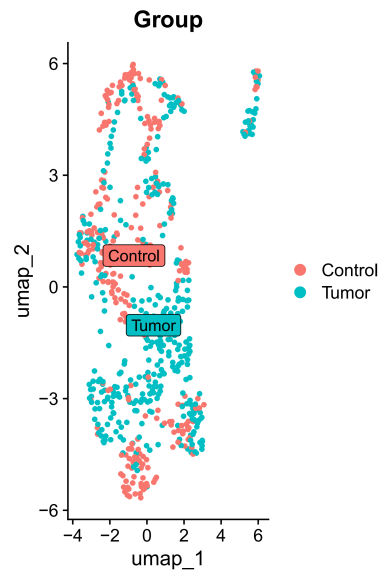

● Control  
● Tumor

PC\_1

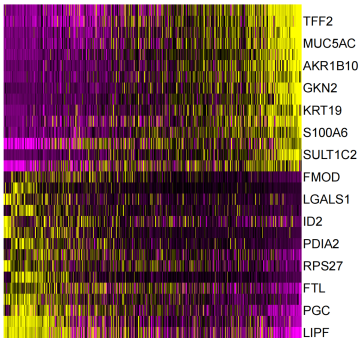

PC\_2

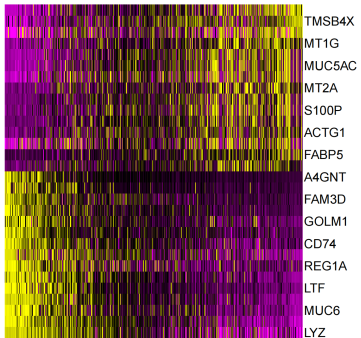

PC\_3

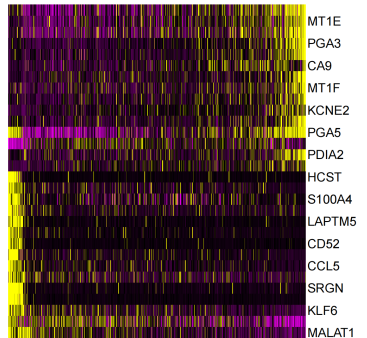

PC\_4

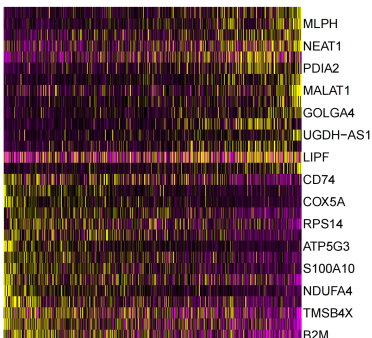

PC\_5

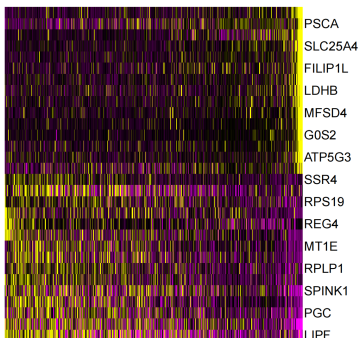

Supplement: S1 Fig — (PDF) [file pone.0338705.s001.pdf]

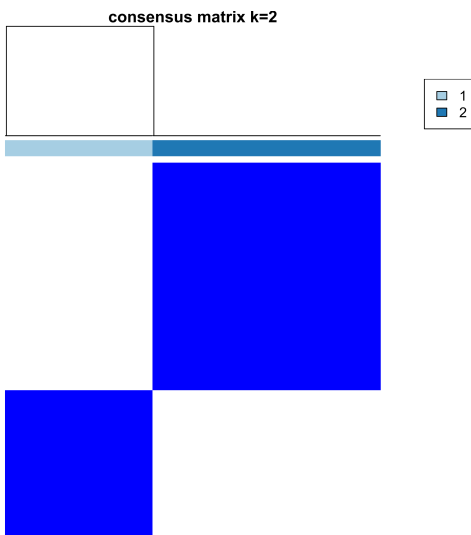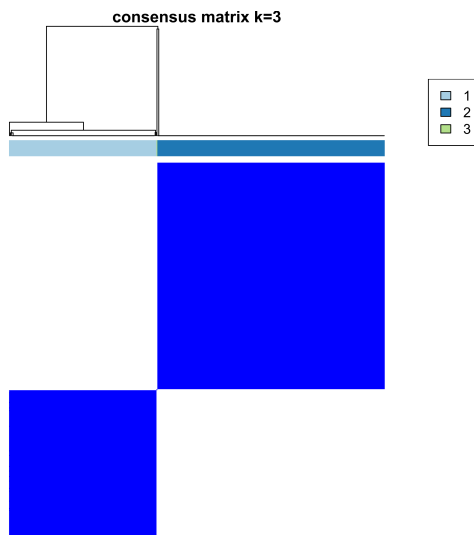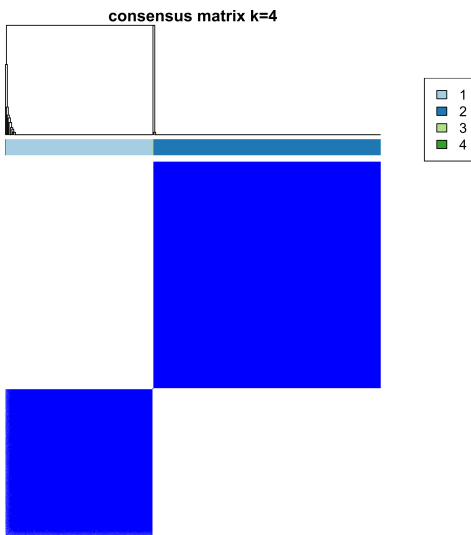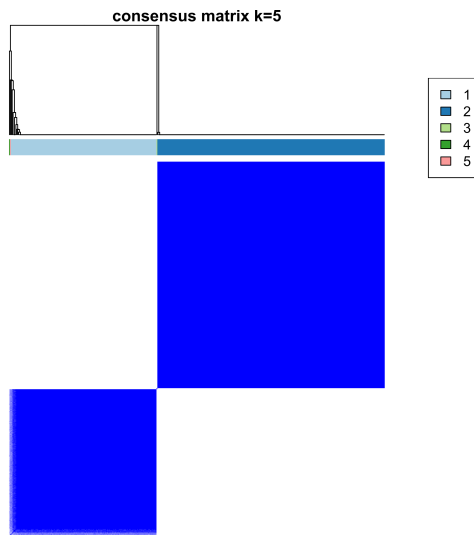

Supplement: S2 Fig — (PDF) [file pone.0338705.s002.pdf]

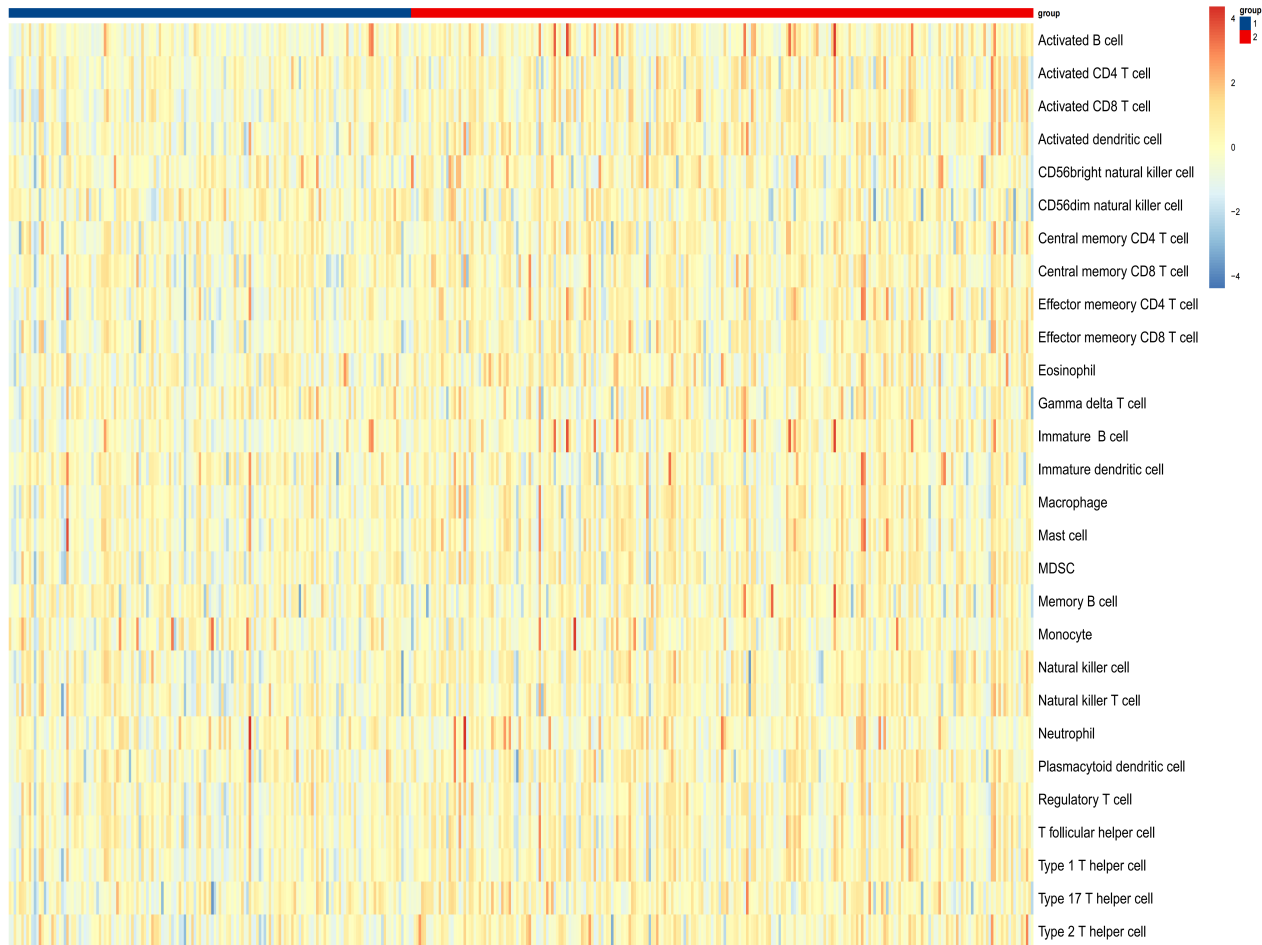

Supplement: S3 Fig — (PDF) [file pone.0338705.s003.pdf]

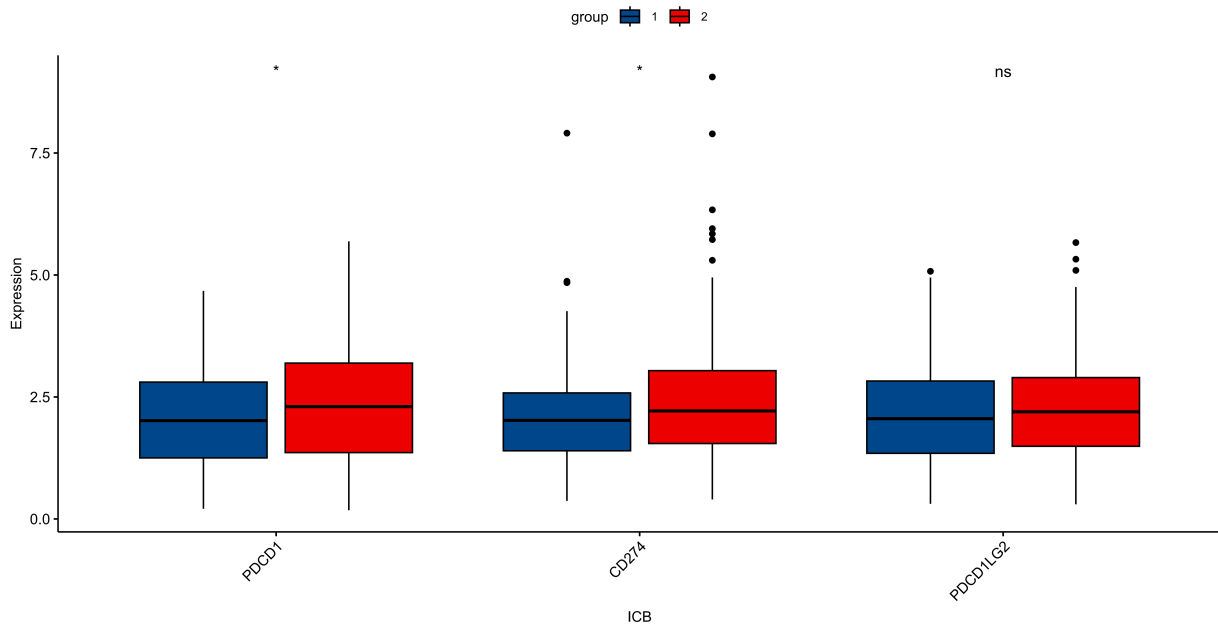

Supplement: S4 Fig — (PDF) [file pone.0338705.s004.pdf]

# B

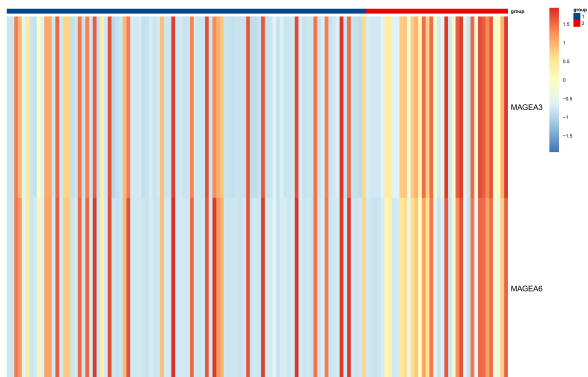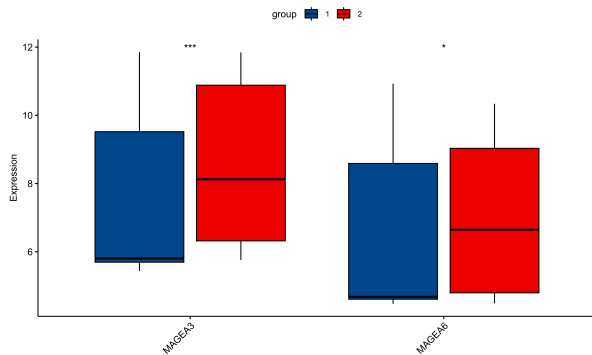

C

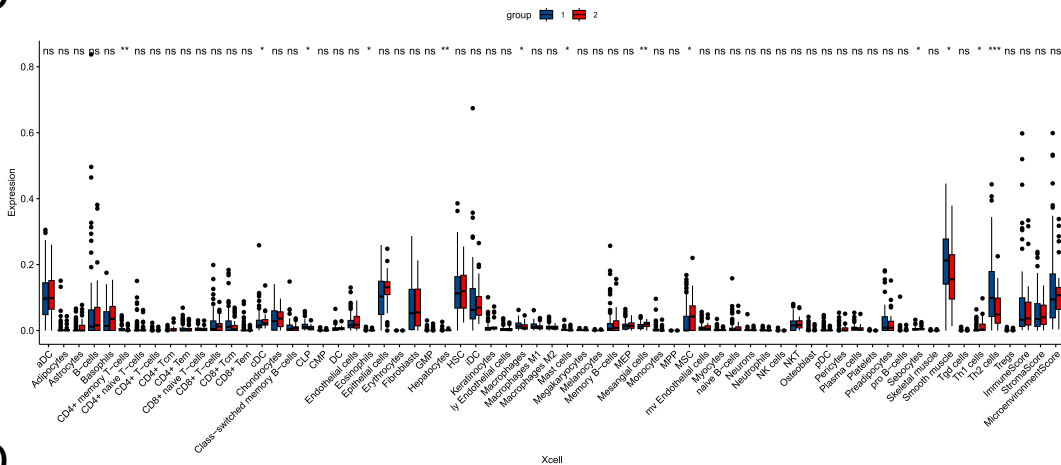

D

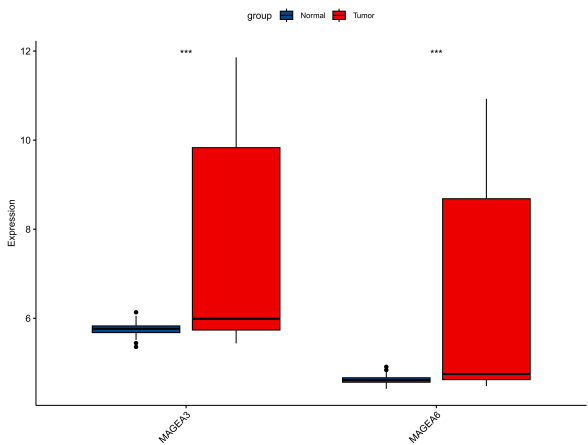

Supplement: S5 Fig — (PDF) [file pone.0338705.s005.pdf]

Top30 Mutation Gene of Group1 (n=161)

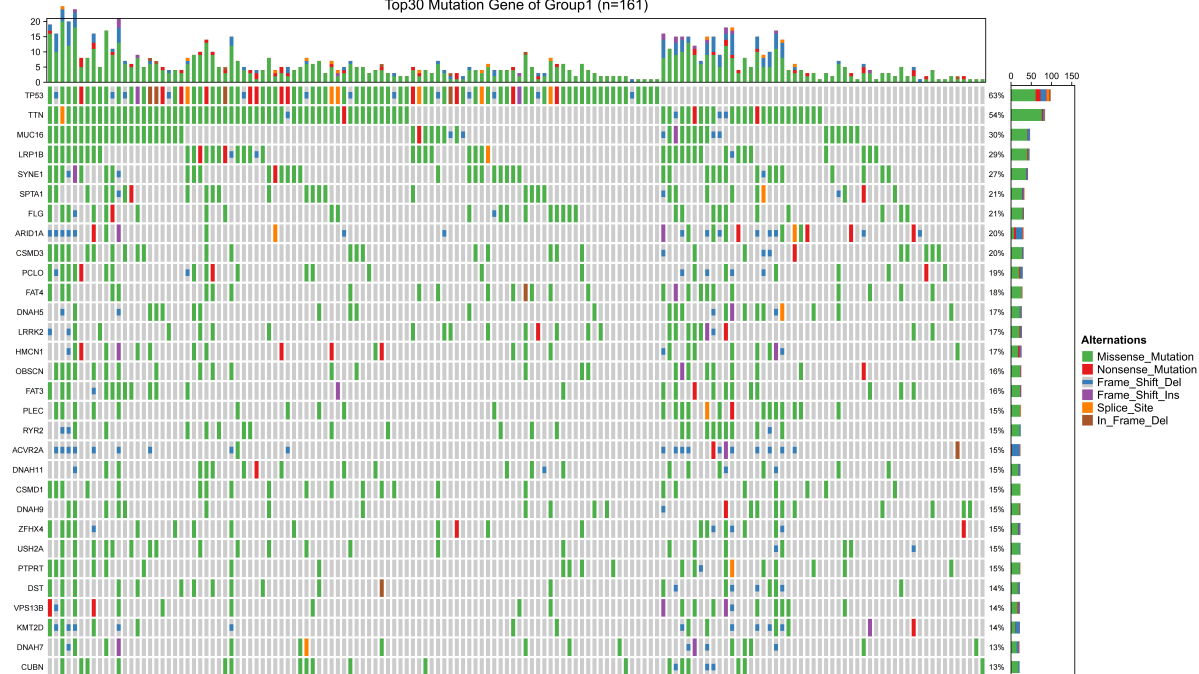

Top30 Mutation Gene of Group2 (n=249)

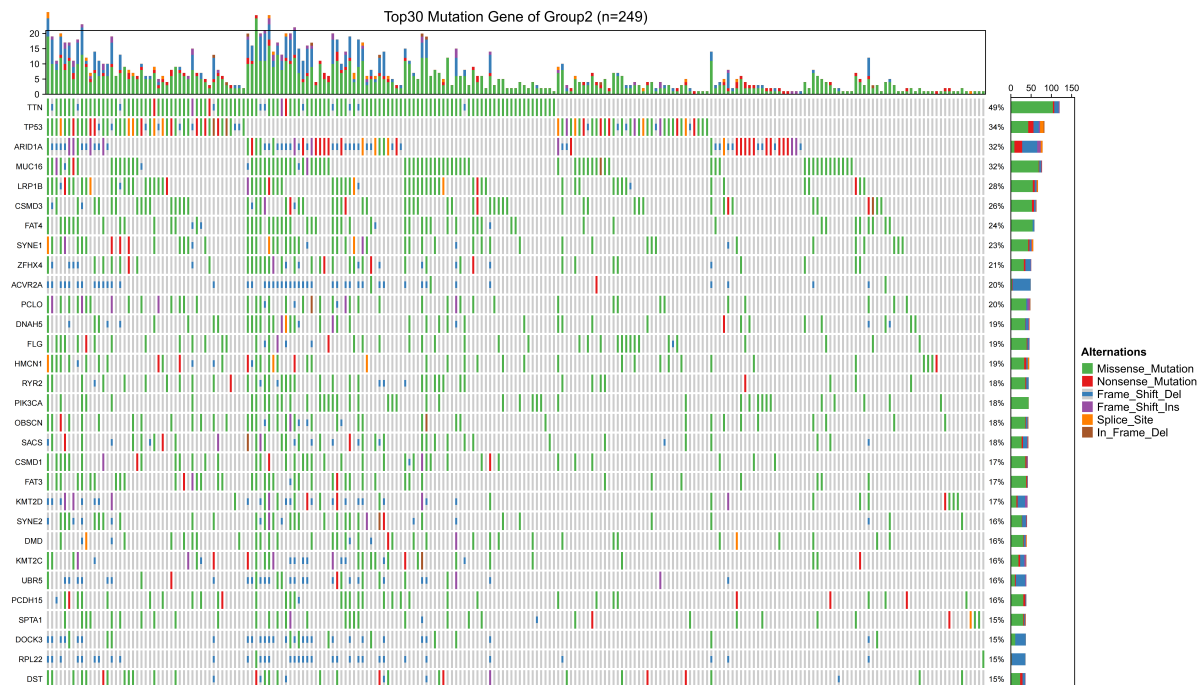

Supplement: S6 Fig — (PDF) [file pone.0338705.s006.pdf]
